# Vancomycin heteroresistance in *Staphylococcus haemolyticus*: elusive phenotype

**DOI:** 10.2144/fsoa-2020-0179

**Published:** 2021-04-09

**Authors:** Yamuna Devi Bathavatchalam, Dhanalakshmi Solaimalai, Anushree Amladi, Hariharan Triplicane Dwarakanathan, Shalini Anandan, Balaji Veeraraghavan

**Affiliations:** 1 Department of Clinical Microbiology, Christian Medical College, Vellore, India; 2 Department of Orthopaedics, Christian Medical College, Vellore, India

**Keywords:** antibiotics, drug development, drug resistance, epidemiology, infectious disease

## Abstract

**Aim:**

To determine the presence of vancomycin heteroresistance in *Staphylococcus haemolyticus*.

**Materials & methods:**

A total of 48 rifampicin-resistant *S. haemolyticus* isolates from bloodstream infections were included. Vancomycin heteroresistance was determined using the population analysis profile-area under curve (PAP-AUC) method. All the isolates were screened for the presence of *mec*A gene, mutations in the *rpo*B gene, staphylococcal cassette chromosome *mec* and multilocus sequence types.

**Results:**

Fifteen isolates were identified as heteroresistant vancomycin-intermediate *S. haemolyticus* using PAP-AUC method. Dual *rpo*B mutations (D471E and I527M) contributed for the rifampicin resistance. The sequence types of heteroresistant vancomycin-intermediate *S. haemolyticus* were highly diverse.

**Conclusion:**

These findings illustrate the potential of *S. haemolyticu*s to develop heteroresistance, which emphasizes the need for routine surveillance of *S. haemolyticus* isolated from intensive care units for infection control practices.

## Lay abstract

The problem of vancomycin-resistant subpopulations of coagulase-negative Staphylococci is rising worldwide; and may adversely affect the response to treatment. This study was conducted to characterize the resistant subpopulations of *Staphylococcus haemolyticus* that cause bloodstream infection, which is largely unexplored. We observed the presence of vancomycin-resistant subpopulations of *S. haemoltyicus*, which are not tested routinely yet should be monitored regularly to potentially improve clinical outcomes.

*Staphylococcus haemolyticus* is an emerging multidrug-resistant (MDR) nosocomial pathogen and is the second most commonly isolated coagulase-negative Staphylococci (CoNS) from blood cultures. *S. haemolyticus* is known to cause bloodstream and device-associated infections in immunocompromised patients [1,2]. *S. haemolyticus* infections are often difficult to treat because of MDR, albeit it possesses fewer virulence factors than *Staphylococcus aureus* [1]. However, studies comparing the pathogenicity traits between clinical and commensal isolates are limited. In Staphylococci, the approach of using marker genes to predict invasiveness can differentiate isolates with different pathogenicity [2]. Similarly in *S. haemolyticus*, resistances to oxacillin and aminoglycosides have been proposed as surrogate markers for invasiveness, while the absence of these traits indicates a commensal flora [2].

Vancomycin has been considered to be the antibiotic of first choice in treating severe infections caused by methicillin-resistant CoNS. However, the increased use of vancomycin has resulted in the development of vancomycin heteroresistance in CoNS. Heteroresistant vancomycin-intermediate CoNS (hVICoNS) occurs with a MIC of 0.5–4 μg/ml, which contains a subpopulation of cells expressing different degrees of resistance to vancomycin and typically present at the frequencies of 10^-4^–10^-6^ [3]. Infections due to hVICoNS present diagnostic challenges and are often difficult to treat. CoNS with a vancomycin MIC of 8–16 μg/ml are characterized as vancomycin-intermediate CoNS (VICoNS). Infections due to hVICoNS have been linked with poor clinical outcomes in patients with bloodstream infections [4–7]. However, a vancomycin MIC-linked outcome has not yet been studied in CoNS.

The prevalence of hVICoNS is underestimated because no standardized methodology has been established for identification. The Clinical and Laboratory Standards Institute (CLSI) recommends MIC determination either by broth microdilution or by the agar dilution method both of which are reported to have suboptimal sensitivity in detecting heteroresistance [8]. The population analysis profile-area under curve (PAP-AUC) method is considered as the gold standard for the detection of heteroresistance [9]. However, this method is labor intensive and may not be suitable for routine use. Detection of a vancomycin heteroresistant subpopulation is challenging, because of multiple and complex molecular mechanisms, phenotype instability, variable vancomycin selective pressure and the lack of specific genetic markers for reliable detection [8].

Studies have linked cell wall thickening, reduced autolysis and decreased surface anionic charges with the development of vancomycin heteroresistance [10]. Studies have also documented a worrying link between the *rpoB* mutation H481Y/N and vancomycin heteroresistance [11,12]. Further, this mutation has been suggested to be a prominent surrogate marker for increased vancomycin resistance in genome wide association studies [13,14]. The H481Y/N mutation not only alters rifampicin susceptibility, but also results in an elevated surface membrane charge which contributes to the cross-resistance between vancomycin and daptomycin [15]. In contrast, a dual *rpo*B mutation (D471E and I527M) conferring vancomycin heteroresistance has been reported in *Staphylococcus epidermidis* [16]. However, the impact of the *rpo*B mutation in the development of heteroresistant vancomycin-intermediate *S. haemoltyicus* (hVISH) has not described.

This study was undertaken: to describe the phenotypic characterization of hVISH, to characterize the mutations in the *rpo*B gene which confer resistance to rifampicin, to investigate the presence of the *ses*I gene and to determine the genotypes of *S. haemolyticus* using staphylococcal cassette chromosome *mec* (SCC*mec*) typing and multilocus sequence typing (MLST).

## Materials & methods

A total of 48 nonrepetitive isolates of rifampicin-resistant *S. haemolyticus* (methicillin resistant: n = 46; methicillin susceptible: n = 2) isolated from patients with hospital-onset (≥48 h of admission) bacteremia, ≥2 consecutive blood cultures positive for *S. haemolyticus* with a time to positivity of <20 h, collected during 2018–2019, were included in the study. Those *S. haemolyticus* isolates from blood cultures on admission or <48 h of admission with a time to positivity of >20 h were excluded from this study. This study was conducted at a 2600-bed tertiary care hospital at the Christian Medical College (Vellore, India). All the isolates were identified as *S. haemolyticus* using matrix-assisted laser desorption/ionization-time of flight mass spectrometer (BioMérieux, Marcy-l'Étoile, France).

## Antimicrobial susceptibility testing

Antimicrobial susceptibility was performed using the disc diffusion method for the following antibiotics: cefoxitin (10 μg), gentamicin (10 μg), erythromycin (15 μg), clindamycin (2 μg), rifampicin (5 μg), trimethoprim-sulfamethoxazole (1.25/23.75 μg), linezolid (30 μg) and minocycline (30 μg). The MIC of vancomycin was determined using the broth microdilution method, as per the CLSI guidelines [17]. Interpretation was carried out according to the CLSI guidelines [18]. Isolates resistant to at least one agent in each of three or more classes of antimicrobials were classified as MDR [19].

## Screening of vancomycin heteroresistance in *S. haemolyticus*

*S. haemolyticus* isolates were preliminarily screened for vancomycin heteroresistance using brain heart infusion agar containing 4-μg/ml vancomycin. The colony-forming unit number per droplet was counted as suggested by Khatib *et al.* [20]. hVISH was determined using the PAP-AUC method as described by Wotton *et al* [9]. All these experiments were repeated twice for each individual strain.

## Molecular characterization of *S. haemolyticus*

All the isolates were screened for the presence of the *mec*A gene and mutations in the rifampicin resistance determining region (RRDR) of the *rpo*B gene [21,22]. SCC*mec* types were determined using a multiplex PCR as described by Milheiriço *et al.* [23]. MLST of *S. haemolyticus* was performed as described by Cavanagh *et al.* [24]. Alleles and sequence types (STs) were assigned using the PubMLST database [25].

## Results

Of the total 48 rifampicin-resistant *S. haemolyticus* isolates, the resistance percentages were as follows: 96% (n = 46) to cefoxitin, 96% (n = 46) to erythromycin, 79.2% (n = 38) to clindamycin, 75% (n = 36) to gentamicin, 69% (n = 33) to trimethoprim-sulfamethoxazole and 4.2% (n = 2) to chloramphenicol. All methicillin-resistant *S. haemoltyicus* isolates were found to have the *mec*A gene. MDR was observed in 98% (n = 47) of the isolates. All the tested isolates were susceptible to vancomycin (MICs: 0.5–4 μg/ml), linezolid and minocycline. All the rifampicin-resistant *S. haemolyticus* isolates were screened for the presence of hVISH. Of these, 46% (n = 22) of the isolates showed growth on brain heart infusion agar containing 4 μg/ml of vancomycin and 31% (15/48) of the isolates were confirmed as hVISH (PAP-AUC ratio: 0.91–1.27) using the PAP-AUC analysis.

**Figure 1.**
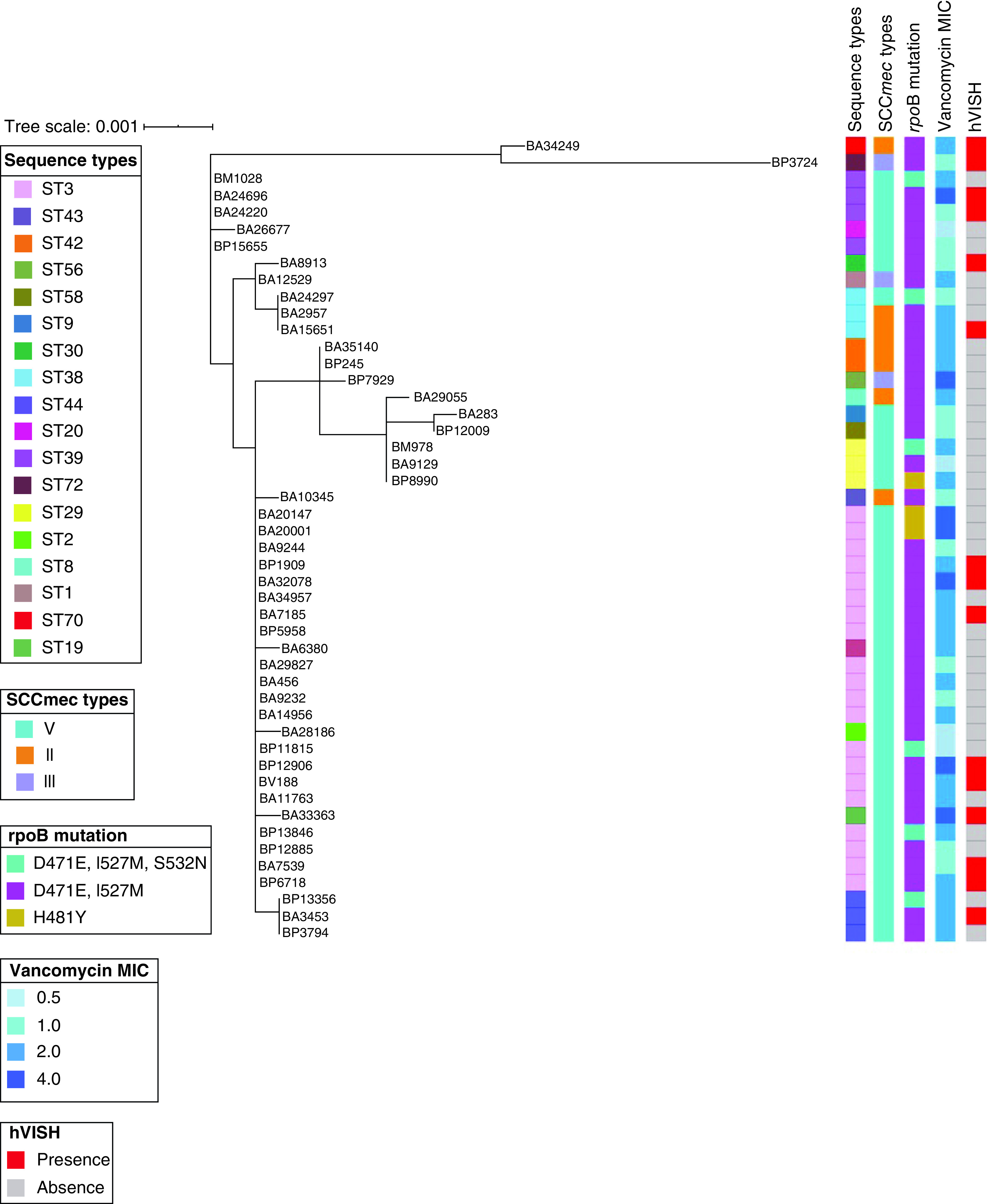
Maximum likelihood phylogenetic tree constructed based on the seven housekeeping genes of *Staphylococcus haemolyticus*. Metadata were annotated with the phylogenetic tree and visualized using iTOL.

Analysis of the mutations in the RRDR of the *rpo*B gene revealed the presence of a double mutation (D471E and I527M) in 81% (n = 39) followed by a triple mutation (D471E, I527M and S532N) in 13% (n = 6) and a single mutation in 6% (n = 3) of the isolates (Figure 1). Notably, all the 15 hVISH isolates had a double mutation (D471E and I527M) in the RRDR of the *rpo*B gene. SCC*mec* typing revealed the presence of three distinct SCC*mec* types (Figure 1). SCC*mec* V was the predominant in 79% (n = 38) of the isolates followed by SCC*mec* II in 15% (n = 7) and SCC*mec* III in 6% (n = 3) of the isolates. The majority of the hVISH isolates, 80% (n = 12) carried SCC*mec* V. MLST analysis of the *S. haemolyticus* (n = 48) revealed high genetic diversity and the isolates belonged to 20 distinct STs (ST1, ST2, ST3, ST8, ST9, ST19, ST20, ST29, ST30, ST38, ST39, ST40, ST42, ST43, ST44, ST56, ST58, ST70 and ST72) (Figure 1). ST3 was predominant and seen in 42% (n = 20) of the isolates followed by ST39 in 8% (n = 4), ST38 and ST44 in 6% (n = 3) of the isolates. Further, hVISH isolates belonged to eight diverse STs (ST3, ST19, ST 30, ST38, ST39, ST44, ST70 and ST72) (Figure 1 & Table 1).

**Table 1.** Molecular characterization of heteroresistant vancomycin-intermediate *Staphylococcus haemolyticus*.

Isolate	*rpo*B mutation	Sequence type	SCC*mec* types	Vancomycin MIC (μg/ml)	PAP–AUC ratio
VB_SH1	D471E, I527M	ST 3	V	2	0.98
VB_SH2	D471E, I527M	ST 3	V	2	1.00
VB_SH3	D471E, I527M	ST 3	V	2	1.03
VB_SH4	D471E, I527M	ST 3	V	2	0.99
VB_SH5	D471E, I527M	ST 3	V	1	0.97
VB_SH6	D471E, I527M	ST 3	V	4	1.08
VB_SH7	D471E, I527M	ST 3	V	4	0.98
VB_SH8	D471E, I527M	ST 19	V	4	1.02
VB_SH9	D471E, I527M	ST 30	V	1	1.05
VB_SH10	D471E, I527M	ST 38	II	2	1.11
VB_SH11	D471E, I527M	ST 39	V	1	1.15
VB_SH12	D471E, I527M	ST 39	V	4	0.99
VB_SH13	D471E, I527M	ST 44	V	2	1.27
VB_SH14	D471E, I527M	ST 70	II	2	0.91
VB_SH15	D471E, I527M	ST 72	III	1	1.21

PAP-AUC: Population analysis profile-area under curve.

## Discussion

Vancomycin heteroresistant CoNS causing bloodstream infections is a growing and unrecognized clinical concern in intensive care patients. Vancomycin heteroresistance and its clinical impact are well studied in *S. aureus* [26,27]. Beside *S. aureus*, heteroresistance to vancomycin has also been reported in *S. epidermidis*, *Staphylococcus capitis*, *S. haemolyticus*, *S. auricularis*, *S. simulans* and *S. warneri* is frequently associated with catheter-related bloodstream infections [28]. Vancomycin heteroresistance in CoNS might impair the clinical response to vancomycin therapy.

Very few studies have reported vancomycin heteroresistance in CoNS. In this study, we demonstrated the presence of hVISH (31%) among the rifampicin-resistant *S. haemolyticus* isolates. Studies have also reported higher rates of vancomycin heteroresistance in CoNS, particularly in *S. capitis* and *S. epidermidis* [29–31]. Certainly, this indicates that hVICoNS can persist within the hospital environment, causing invasive infections, and is much more prevalent than previously assumed. It is well known that hVISA is recognized to cause a longer duration of bacteremia and vancomycin treatment failure is 2.37-times higher than vancomycin-susceptible *S. aureus* [32,33]. Meanwhile, the clinical impact of the hVICoNS is not clear.

Mutations in *rpo*B have been reported to be associated with the development of hVISA (H481Y/N) and vancomycin heteroresistance in MDR *S. epidermidis* (D471E and I527M). Similarly, in the present study, all the hVISH isolates had dual mutations D471E with I527M in the *rpo*B gene. Interestingly, *rpoB* mutation-driven vancomycin heteroresistance has been reported in *S. aureus* [34,35], *S. epidermidis* [16] and *S. capitis* [7]. It is interesting to note that the occurrence of dual *rpo*B mutations (D471E and I527M) contributes to rifampicin resistance in *S. haemolyticus*. Furthermore, combination therapy of vancomycin with rifampicin is likely to promote the development of cross-resistance between these agents. We postulate that rifampicin resistance in *S. haemolyticus* may increase the risk for the development of vancomycin heteroresistance and treatment failure. However, additional experiments and clinical outcome-based observations are required to confirm this hypothesis.

Clonal spread of heteroresistant CoNS in the neonatal intensive care unit (NICU) has been described with diverse sequence types [6]. These studies have emphasized the potential of hVICoNS for cross-transmission in NICU. However, molecular marker for the precise detection of vancomycin heteroresistance in S. haemolyticus has not documented. Notably, vancomycin heteroresistance is an intrinsic feature in *S. capitis* and the clonal spread is restricted to NICUs [36]. Meanwhile, in *S.* *epidermidi*s, majority of the vancomycin heteroresistant strains belongs to two multi-drug lineages ST2 and ST23 [37]. However, in the present study, genetically diverse hVISH belonging to 20 distinct STs was observed. Collectively, successful establishment of multiple hVICoNS clones may increase the potential for rapid dissemination in intensive care units.

## Conclusion

In conclusion, vancomycin heteroresistant CoNS causing bloodstream infection is a growing concern. Dual (D471E and I527M) or triple (D471E, I527M and S532N) mutations contribute for the development of rifampicin resistance in *S. haemolyticus*. The present finding revealed that vancomycin heteroresistance in *S. haemolyticus* is high (31%), which implies the potential reduction in vancomycin susceptibility. The hVISH isolates have susceptible vancomycin MICs and cannot be detectable with conventional susceptibility testing methods; a standardized method is required to detect vancomycin heteroresistance in CoNS in clinical settings. Screening methods are recommended and should be considered to improve clinical outcome in high-risk patients. Vancomycin heteroresistance in CoNS is associated with the risk of poor clinical outcomes which has yet to be explored. Further, surveillance studies are required to understand the true prevalence of vancomycin heteroresistance in CoNS.

## Future perspective

Vancomycin heteroresistance in CoNS is of clinical concern and is being increasingly reported across the globe. Although the clinical impact of vancomycin heteroresistance in *S. haemolyticus* is relatively unexplored. Further, studying the impact of vancomycin MICs on the clinical outcome of patients with persistent infections with *S. haemolyticus* would be helpful. Whole genome sequencing data on hVISH will help to decipher the candidate genes and the mutations involved in the development of vancomycin heteroresistance.

**Summary points**

- Vancomycin has been considered to be the antibiotic of first choice in treating severe infections caused by methicillin-resistant coagulase-negative Staphylococci (CoNS).
- Heteroresistance to vancomycin is being increasingly reported in CoNS.
- Studies have documented poor clinical outcomes in patient with heteroresistant vancomycin-intermediate CoNS infections.
- *Staphylococcus haemolyticus* is an emerging multidrug-resistant nosocomial pathogen and is the second most commonly isolated CoNS from blood cultures.
- Studies have documented a worrying link between the rpoB mutation H481Y/N and vancomycin heteroresistance.
- In this study, 96% of the *S. haemolyticus* isolates were resistant to cefoxitin and multidrug-resistant was seen in 98% (n = 47) of the isolates.
- Vancomycin heteroresistance was seen in 31% of the tested rifampicin-resistant *S. haemolyticus* isolates. These isolates have susceptible vancomycin MICs (0.5–4 μg/ml) and cannot be detectable with conventional susceptibility testing methods.
- Rifampicin resistance is mainly mediated by the presence of a double mutation (D471E and I527M).
- Multilocus sequence typing analysis of the *S. haemolyticus* (n = 48) revealed high genetic diversity and the isolates belonged to 20 distinct sequence types (ST1, ST2, ST3, ST8, ST9, ST19, ST20, ST29, ST30, ST38, ST39, ST40, ST42, ST43, ST44, ST56, ST58, ST70 and ST72).
- Screening methods are recommended and should be considered to improve clinical outcome in high-risk patients. Further, surveillance studies are required to understand the true prevalence of vancomycin heteroresistance in CoNS.
